# EFFECTS OF SOCIAL ISOLATION ON THE WELL-BEING AND COPING MECHANISMS OF OLDER ADULTS IN BISHOFTU TOWN, ETHIOPIA

**DOI:** 10.1093/geroni/igae098.3229

**Published:** 2024-12-31

**Authors:** Messay Kotecho, Abraham Kassay, Margaret Adamek

**Affiliations:** Addis Ababa University, Addis Ababa, Adis Abeba, Ethiopia; Addis Ababa University, Addis Ababa, Adis Abeba, Ethiopia; Indiana University Indianapolis, Indianapolis, Indiana, United States

## Abstract

Research concerning older adults is gaining attention in Ethiopia. Nevertheless, previous research has disregarded the issue of social isolation among elders in Ethiopia. The prevalence of social isolation among older adults is now a significant concern worldwide due to its impact on both individuals and society at large. This study examined how social isolation affects the well-being of older adults and their coping mechanisms, focusing specifically on the experiences of older adults in Bishoftu Town, Ethiopia. A phenomenological approach was used to investigate the experiences of 10 older adults (5 male and 5 female) aged 60 and above, purposefully selected for in-depth interviews and observations. The results indicate that participants are increasingly susceptible to social isolation due to factors such as negative perceptions of aging, loss of meaningful relationships, economic hardships, and age-related health issues. Consequently, elders experience a reduction in social connections, health issues, psychological distress, and challenges in meeting basic needs. Despite these obstacles, older adults have developed various coping strategies, including engaging in prayer and spiritual practices, depending on intergenerational support, participating in traditional savings associations (Idir), and sleeping excessively. Nonetheless, many participants express feelings of pessimism, weakness, lack of affection, and a sense of worthlessness about their future. Additionally, participants report facing disengagement and a loss of social ties alongside grappling with severe poverty and inadequate access to healthcare services. The study underscores the urgent need to bolster intergenerational solidarity and social capital within the community to tackle the multifaceted challenges confronting older adults in Ethiopia.

